# PINP: A New Method of Tagging Neuronal Populations for Identification during *In Vivo* Electrophysiological Recording

**DOI:** 10.1371/journal.pone.0006099

**Published:** 2009-07-07

**Authors:** Susana Q. Lima, Tomáš Hromádka, Petr Znamenskiy, Anthony M. Zador

**Affiliations:** 1 Cold Spring Harbor Laboratory, Cold Spring Harbor, New York, United States of America; 2 Champalimaud Neuroscience Programme, Instituto Gulbenkian de Ciência, Oeiras, Portugal; 3 Cold Spring Harbor Laboratory, Watson School of Biological Sciences, Cold Spring Harbor, New York, United States of America; Yale School of Medicine, United States of America

## Abstract

Neural circuits are exquisitely organized, consisting of many different neuronal subpopulations. However, it is difficult to assess the functional roles of these subpopulations using conventional extracellular recording techniques because these techniques do not easily distinguish spikes from different neuronal populations. To overcome this limitation, we have developed PINP (Photostimulation-assisted Identification of Neuronal Populations), a method of tagging neuronal populations for identification during *in vivo* electrophysiological recording. The method is based on expressing the light-activated channel channelrhodopsin-2 (ChR2) to restricted neuronal subpopulations. ChR2-tagged neurons can be detected electrophysiologically *in vivo* since illumination of these neurons with a brief flash of blue light triggers a short latency reliable action potential. We demonstrate the feasibility of this technique by expressing ChR2 in distinct populations of cortical neurons using two different strategies. First, we labeled a subpopulation of cortical neurons—mainly fast-spiking interneurons—by using adeno-associated virus (AAV) to deliver ChR2 in a transgenic mouse line in which the expression of Cre recombinase was driven by the parvalbumin promoter. Second, we labeled subpopulations of excitatory neurons in the rat auditory cortex with ChR2 based on projection target by using herpes simplex virus 1 (HSV1), which is efficiently taken up by axons and transported retrogradely; we find that this latter population responds to acoustic stimulation differently from unlabeled neurons. Tagging neurons is a novel application of ChR2, used in this case to monitor activity instead of manipulating it. PINP can be readily extended to other populations of genetically identifiable neurons, and will provide a useful method for probing the functional role of different neuronal populations *in vivo*.

## Introduction

Much of what we know about how the intact mammalian brain encodes information, and about the relation between neural activity and behavior, we owe to extracellular recording techniques. Extracellular recording is one of the main techniques available for studying the spike trains of individual neurons *in vivo*, particularly in behaving animals. However, an important limitation of extracellular recording is that it provides little information about the identity of the neurons generating the spike trains, and therefore provides limited insight into the function of different neuronal populations. Neurons within an area can be excitatory or inhibitory; they can receive input from and project to different brain areas; and they can express different complements of channels, neurotransmitters, receptors, and other molecules. A general method for determining these and other characteristics of neurons recorded *in vivo* would be valuable for establishing the functional role of different neuronal populations.

Historically, two main approaches have been used to identify specific neuronal populations during extracellular recording. First, spike width has been used to distinguish excitatory from inhibitory neurons, although interpretation is complicated because not all interneurons have narrow spikes and some narrow spiking neurons are not interneurons [1], [2], [3], [4], [5]. Second, neurons projecting from area X to area Y can be identified by antidromic activation of axons in area Y combined with simultaneous recordings in area X. Although this approach has provided important insights into how circuits are organized [6], [7], [8], [9], [10], antidromic stimulation is technically challenging.

A method for identifying neurons based on expression of a genetically-encoded reporter would provide a powerful and more general approach for probing the role of different neuronal populations in brain circuits. Whole cell patch clamp can be combined with histological, immunochemical and molecular methods (e.g.( [11])) to establish molecular expression patterns, but these techniques offer low yield in vivo. Green fluorescent protein (GFP) has been widely used as a marker of specific cell populations for over a decade. Whole cell patch clamp can be combined with fluorescence imaging to target GFP-labeled neuronal populations [12], but this is technically challenging in vivo. More recently, genetically-engineered variants of GFP that report neuronal activity have been developed, notably calcium sensors such as GCaMP [13] and the pH-sensors such as pHluorins [14], but even the best of these indicators have yet to reach the resolution necessary to detect single spikes in single neurons [15] and their use in vivo is limited.

**Figure 1:**
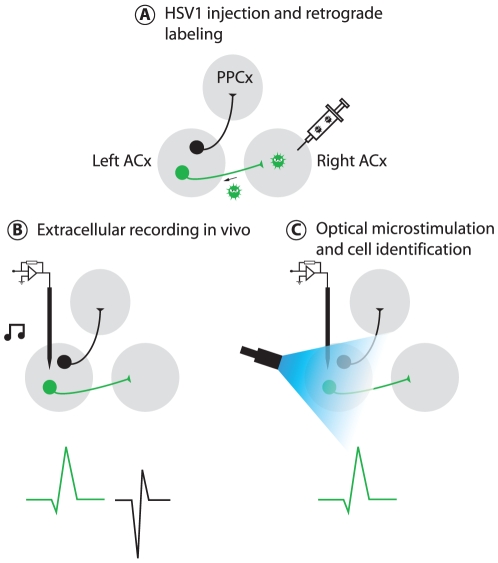
Identifying ChR2-tagged neurons in vivo. Photostimulation-assisted identification of neuronal populations: general method for identifying neuronal populations during *in vivo* electrophysiological recordings. (A) ChR2 expression is restricted to distinct neuronal populations using methods that allow targeting of genetically identifiable populations of neurons (more detail in text). (B) Spikes from ChR2-positive (*green*) and ChR2-negative (*black*) single units are recorded extracellularly during a normal *in vivo* experiment, for example in response to sound stimulation. (C) ChR2-positive units are identified on the basis of their response to a flash of blue light.

We have therefore developed Photostimulation-assisted Identification of Neuronal Populations (PINP), a general method of tagging genetically-defined populations of neurons for identification during *in vivo* electrophysiological recordings, which is schematically represented in Figure 1. The tag is channelrhodopsin-2 (ChR2), a light-gated cation-selective channel originally cloned from algae [16] and adapted to mammalian neurons [17]. Expression of ChR2 can be genetically restricted to populations of neurons in the same way as other genetically encoded reporters (Figure 1A). In contrast to other indicators like GFP or calcium sensors, however, which must be detected optically—a technical challenge *in vivo*, particularly for deep brain structures—ChR2 can be detected electrophysiologically *in vivo*: illumination of ChR2-tagged neurons with a brief flash of blue light triggers a short latency reliable action potential (Figure 1B and C). PINP represents a novel application of ChR2, because instead of using it to manipulate or perturb neuronal activity we are using it to monitor activity.

In this report we demonstrate that ChR2-positive neurons can be reliably distinguished from ChR2-negative neurons *in vivo* using extracellular recording methods. To demonstrate the generality of PINP, we describe two different methods based on viral-mediated gene transfer. With both methods, ChR2 expression was restricted to the neuronal populations of choice by exploiting features known to underlie neuronal diversity. In the first method, we labeled a population of cortical neurons—mainly fast-spiking interneurons—by using adeno-associated viral (AAV) gene transfer in a transgenic mouse line [18] in which the expression of Cre recombinase was driven by the parvalbumin promoter. In the second method, we labeled populations of excitatory neurons based on projection target by using herpes simplex virus 1 (HSV1), which is efficiently taken up by axons and transported retrogradely. Using these two methods to label distinct populations of auditory cortex neurons, we show that responses in tagged neurons are different from those in un-tagged neurons.

## Results

To test the feasibility and generality of using ChR2 as a physiological tag, we expressed ChR2 in two distinct neuronal populations within the rodent auditory cortex. In one set of experiments, we used AAV to restrict ChR2 expression to a population of inhibitory interneurons expressing parvalbumin in the mouse [18]. In a second set of experiments, we used HSV1 to restrict ChR2 expression to a population of neurons in the rat based on their projection to a particular target. Using these two approaches we were able to tag two populations of auditory cortex neurons and identify them during *in vivo* extracellular electrophysiological recordings.

The results are organized as follows. First we demonstrate expression of ChR2 in either PV-expressing interneurons or callosally-projecting pyramidal neurons. Second we show that we can photostimulate and identify PV-positive neurons in the mouse auditory cortex. Third we show that we can photostimulate and identify layer 3 and 5 cortical neurons that project to the contralateral auditory cortex, and that this population is functionally distinct from the unlabeled population of cells in the rat auditory cortex.

### Virally-mediated expression of ChR2-YFP in distinct populations of auditory cortex neurons

#### PV interneurons

**Figure 2:**
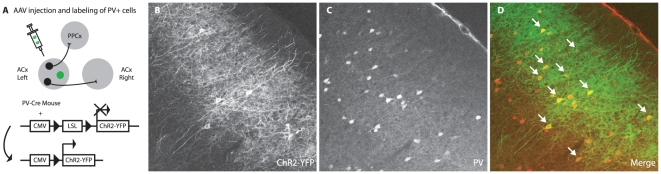
Viral-mediated expression of ChR2-YFP into a class of inhibitory interneurons in the mouse auditory cortex. (A) Neurons within the rodent auditory cortex can be excitatory or inhibitory. To express ChR2 in inhibitory parvalbumin expressing neurons of the mouse auditory cortex, we injected AAV carrying floxed ChR2-YFP in the left auditory cortex of PV-Cre mice, which express Cre recombinase only in fast-spiking interneurons. Although the virus can infect any cell, ChR2 is expressed only in PV-positive neurons. (B) Confocal micrograph of a section including the mouse primary auditory cortex shows fluorescence in cells expressing ChR2-YFP. (C) To test for PV specificity, the section was treated with an antibody against PV and counterstained with a red fluorescent dye. (D) Merging of the two channels shows that cells expressing YFP also counterstain for PV (merged cells show as yellow). Note that all ChR2+ (green) cells are also PV-positive (red) (i.e. there are only a few false positives), but that not all red PV-positive cells express ChR2.

We first restricted expression of ChR2 to one particular type of inhibitory interneuron, the fast spiking basket cells that express parvalbumin (PV). To restrict expression, we used a new variant [19] (Figure 2A) of the binary Cre/LoxP expression system [20], in which a target DNA sequence of interest is flanked by specific short sequences of DNA (“loxP” sites). The loxP site pair is the target of an enzyme, Cre-recombinase, which removes the sequences between the sites. We then engineered an AAV in which expression of ChR2 expression was inhibited in all cell types by a transcriptional insulator (a “STOP” sequence) flanked by loxP sites (LoxP-STOP-LoxP, or LSL) upstream of the coding sequence; expression of ChR2 occurs only if the STOP insulator is excised, which occurs only in cells expressing Cre (Figure 2A). We injected this virus into the auditory cortex of a transgenic mouse line in which Cre expression was driven by the promoter for PV and therefore limited to PV-positive interneurons [18]. Thus expression of ChR2 should occur only in neurons expressing Cre-recombinase (i.e. PV-interneuron) and infected with the LSL-AAV, since only in these neurons will the STOP sequence be excised.

We confirmed PV-Cre mediated ChR2-YFP expression using histological methods (Figure 2B, C and D). We injected AAV-LSL-ChR2 virus into the left auditory cortex of PV-Cre mice and prepared coronal brain slices at least 2 weeks post-infection. To determine the specificity of ChR2-YFP expression (Figure 2B) the sections were incubated with anti-PV antibody and counterstained with secondary red fluorescent antibody (Figure 2C). Figure 2D shows a typical example in which of 92 YFP positive cells observed, PV staining was co-localized in 89 (97% of cells). Thus using ChR2-YFP as a marker for PV expression, the false positive rate was only 3% (*i.e.* only 3% of ChR2-YFP expressing neurons did not express PV), indicating that ChR2-YFP expression provided a reliable tag for parvalbumin-expressing interneurons.

#### Callosal neurons

**Figure 3:**
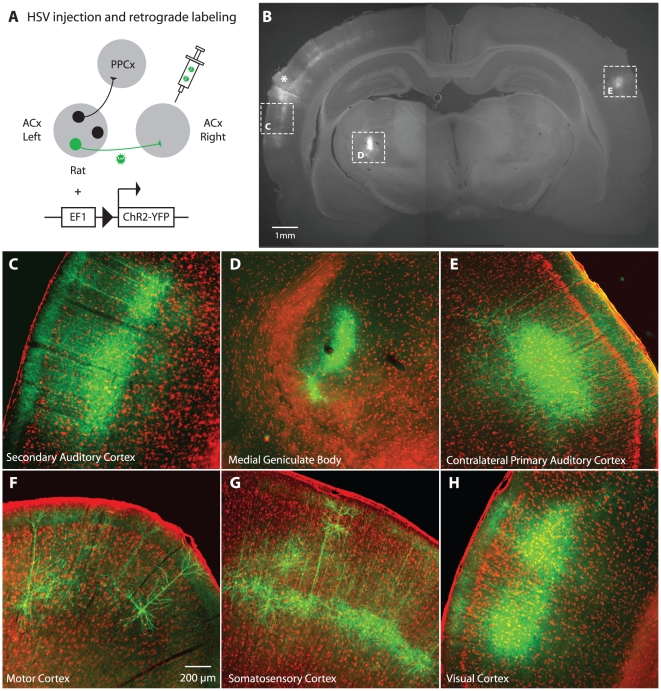
Viral mediated retrograde labeling of neurons projecting to the primary auditory cortex. (A) To tag neurons based on projection pattern, HSV1 expressing ChR2-YFP was injected into the right auditory cortex. Ten days later, coronal brain sections were made to assess infected cells (*green*); sections were counterstained with *red* fluorescent Nissl substance to stain neurons. (B) Coronal section showing the site of injection (*asterisk*) and ipsilateral secondary auditory cortex (*above left box*). Staining can also be seen in the auditory thalamus (*middle box*) and contralateral auditory cortex (*right box*). Retrogradely labeled areas include: (C) nearby ipsilateral secondary auditory cortex; (D) ipsilateral medial geniculate (auditory) thalamus; (E) neurons in layers 3 and 5 of the contralateral primary auditory cortex; (F) motor cortex; (G) somatosensory cortex; (H) visual cortex.

We next targeted ChR2 expression based on anatomical projection pattern. Pyramidal neurons in the auditory cortex project to multiple brain regions. We engineered an attenuated strain of HSV1, a neurotropic virus which travels retrogradely through axons, to restrict ChR2 expression to a specific population of these output neurons (Figure 3A). The particular strain of HSV1 we used has multiple genomic deletions which render it replication-defective and low toxicity [21]. In this way we could target subsets of neurons within the rat auditory cortex based on their axonal projection to the site of the HSV1 injection.

We confirmed HSV1-mediated ChR2-YFP expression using histological methods (Figure 3B to H). We injected the HSV1 into the left auditory cortex and prepared coronal brain slices 10 days later (Figure 3B). We counterstained sections with red fluorescence-conjugated Nissl substance to reveal the laminar structure of the cortex. We observed dense expression of ChR2-YFP in layers 3 and 5 neurons contralateral to the injection site; neurons in these layers project their axons to the contralateral auditory cortex via the corpus callosum (Figure 3E), [22]. In addition to the contralateral auditory cortex, we observed expression in the other subcortical and cortical areas that project to the auditory cortex (Figure 3, boxes C and D, F–H) [23]. These results thus confirm that the HSV1 construct can be used to deliver ChR2-YFP to specific pathways projecting to the auditory cortex.

#### In vivo photostimulation of fast-spiking interneurons in the auditory cortex

**Figure 4:**
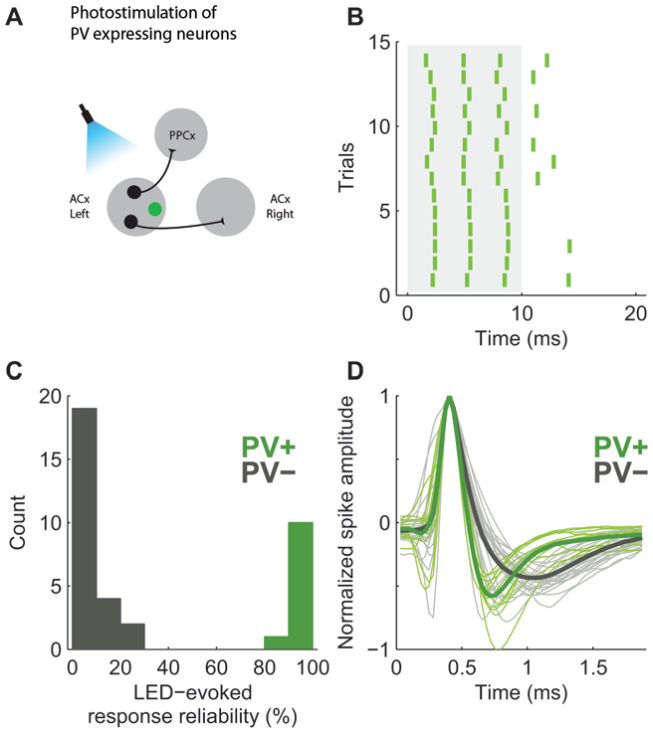
In vivo photostimulation of parvalbumin expressing auditory cortex neurons. (A) PV expressing neurons in the mouse auditory cortex, labeled with the binary Cre-AAV system, were tagged with ChR2 (green). (B) Spike rasters of a well isolated single unit that responded to light activation in the mouse auditory cortex. Light was on from 0 to 10 ms. (C) Reliability of light-evoked responses in all the cells recorded in the mouse auditory cortex. Reliability was computed as the fraction of trials in which the firing rate within the 40 ms after the start of the light pulse was greater than within the 40 ms immediately preceding the light pulse. (D) Action potentials originated from ChR2-expressing neurons were narrower than spikes originated from the rest of the population (*green* - ChR2 positive, *gray* – unlabeled cells).

After establishing that we could use the binary Cre-viral system to express ChR2 in parvalbumin expressing neurons, we next tested whether we could detect light triggered activity in this population of tagged neurons. We injected AAV-LSL-ChR2-YFP in area A1 of the left primary auditory cortex of PV-Cre mice. To activate ChR2-tagged neurons *in vivo*, we used a simple photostimulation system consisting of a blue LED (477 nm) coupled to an optical fiber (1 mm diameter) positioned with the help of a micromanipulator over the exposed auditory cortex of anesthetized mice (as shown in the cartoons, Figure 4A) (see *Experimental Procedures for details*). We interleaved photostimulation (10 ms LED pulses) with white noise acoustic stimulation (35 ms duration, 70 dB) presented at 0.67 Hz. We searched for ChR2-expressing (and hence PV-expressing) neurons by slowly advancing a tungsten extracellular recording electrode through the auditory cortex until each light flash elicited a reliable short-latency (2.8±1.3 ms; mean±SD) response consisting of one or more spikes (Fig. 4B). We also recorded from a control group of cells not responsive to light flashes (Figure 4C).

PV neurons correspond to fast-spiking basket cells, and can sometimes be distinguished during extracellular recordings on the basis of their narrower spikes [4], [5], [24], [25]. We therefore compared spike widths between the light responsive ChR2-expressing population and the non-responsive control population. The average waveforms for the light-responsive population were narrower, consistent with the interpretation that ChR2 labeled narrow spiking PV neurons (Figs. 4D). Furthermore, the spike waveforms triggered by light stimulation were indistinguishable from those triggered by acoustic stimulation (*data not shown*), indicating that triggering spikes with ChR2 was not perturbing waveform shape. These results show that LED-evoked responsiveness can be used to identify PV-expressing interneurons expressing ChR2 in the cortex.

To test whether ChR2-tagged PV-positive neurons differed in their auditory responsiveness from nearby untagged neurons selected randomly, we assessed the neural response elicited in each population by white noise bursts. Because of their name—parvalbumin is a marker for the so-called “fast-spiking” population of interneurons—and previous *in vivo* results [5], we expected that ChR2-tagged neurons would be more responsive. Surprisingly, sound-evoked firing rates in tagged neurons (median 6.9 Hz, N = 11) were not different from those in untagged neurons (12 Hz, p = 0.2268, N = 25), nor was there a significant difference in the fraction of sound responsive neurons between the two populations (PV-positive 5/11, PV-negative 13/25, p = 0.50). Spontaneous firing rates were significantly higher in the untagged population (median 1.5 Hz) than in the tagged population (median 0.65 Hz, Mann-Whitney U test p = 0.0110). Thus the population of fast-spiking tagged interneurons appeared to be similar to nearby untagged neurons.

#### In vivo photostimulation of callosally-projecting neurons

**Figure 5:**
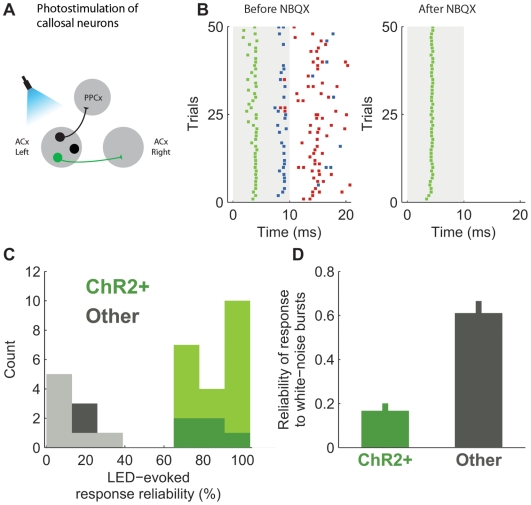
In vivo photostimulation of callosally projecting auditory cortex neurons. ChR2 expression in rat auditory cortex neurons can be used to tag and identify this neuronal population during *in vivo* recordings. (A) Callosally projecting neurons in the rat auditory cortex were labeled with ChR2 via retrogradely HSV1-mediated transfection; (B) Light-evoked activity of three well-isolated single units recorded simultaneously is shown as rasters. Each unit is color-coded (*green, blue, red*). After blocking fast glutamate (AMPA) receptors (left) with the selective antagonist NBQX, only the shortest latency unit (*green*, right) continued to show light-activated activity. This indicates that activity in the other two units was indirect, *i.e.* synaptic, and therefore blocked by NBQX. (C) Population histogram showing that ability to follow light flashes at higher repetition rate (5 Hz, ISI = 200 ms, 10 ms LED pulses) cleanly separated recordings into two classes, which we interpret as direct (ChR2-positive; dark green: single units responding after NBQX application) and synaptic (ChR2-negative; dark gray: single units not responding after NBQX simulation; light gray: multiunit recordings). The x-axis shows the spiking response reliability, computed as the fraction of trials in which the firing rate in 40 ms after the start of the second pulse of 5 Hz LED trains was greater than in the 40 ms immediately preceding the pulse. (D) Callosally projecting neurons (ChR2+) are non-responsive to white noise stimulation, showing different response reliability from the average population (Other). Reliability was computed as the fraction of trials in which the firing rate in 40 ms after sound onset was greater than in 40 ms immediately preceding the sound. Error bars show standard error of the mean.

Injection of HSV1-ChR2-YFP into the auditory cortex of the rat led to a high density of ChR2-YFP expression in layers 3 and 5 of the contralateral auditory cortex (Figure 3). We reasoned that these callosally-projecting neurons would be a suitable target for photostimulation. We therefore injected HSV1-ChR2-YFP into the right primary auditory cortex, and recorded light-evoked activity in area A1 of the contralateral (left) cortex at least 8 days after injection. To record and photostimulate rat layer 3 and 5 pyramidal neurons whose axons project to the contralateral cortex we used a setup similar to the one described for the parvalbumin expressing neurons in the mouse (as depicted in Figure 5, see *Experimental Procedures for details*). As observed in the previous experiments with PV-neurons, a brief flash of light elicited action potentials (Figure 5B). The average latency of the first light-evoked spike was much lower than the latency of the first sound-evoked spike recorded in the auditory cortex (6±2 ms vs 20±5 ms; mean±SD), and depended on the intensity and duration of the light pulse. In some cases, more intense or longer-lasting flashes triggered multiple spikes. Comparison of spontaneous action potentials to light evoked action potentials revealed that the two were similar (*data not shown*). These results demonstrate HSV1 mediated expression of ChR2 can be used to generate spiking activity.

Because of local excitatory connectivity within the auditory cortex, we were concerned that ChR2-mediated activation of callosally-projecting neurons could lead to indirect activation of synaptically connected neurons in the illuminated area. Our goal was to tag a specific population, therefore indirect activation would confound the identification of putative ChR2-expressing neurons. Indirect activation was less likely to arise in the identification of PV-positive neurons described above because PV-positive neurons are inhibitory, so activation by light tended to suppress activity in other synaptically connected neurons.

We first confirmed that our recordings included both directly and indirectly activated neurons. We compared light-evoked responses before and after application to the surface of the cortex of the AMPA receptor blocker NBQX, which blocks most excitatory transmission in the cortex. An example recording experiment is shown in Figure 5B. Prior to application, we isolated three different neurons simultaneously on a single tetrode, each responding with a different latency. As expected, NBQX application blocked sound-evoked responses (*not shown*). After application, a light flash evoked spikes only in the shortest latency unit (Figure 5B), indicating that the other two units were indirectly activated. Interestingly, the latency of this remaining unit became both longer and more precise, possibly because even in the directly activated unit spike initiation was sometimes augmented by indirect synaptic activation, presumably arising from ongoing activity found *in vivo*.

Although reliable light-evoked activity after blockade of excitatory transmission is definitive evidence for direct activation and therefore that the neuron expresses ChR2, applying a synaptic blocker after each recording would be cumbersome. We therefore tested a second method for distinguishing direct from synaptic activation, based on the observation that sensory stimuli do not reliably elicit spikes at high (above 5–10 Hz) repetition rates in cortical neurons [26], [27], [28], whereas it has been shown that direct activation with ChR2 can elicit spikes at high repetition rates [17] and so we hypothesized that only directly activated ChR2-tagged neurons *in vivo* would follow high-frequency stimulus trains reliably. Across the population, we found that all well-isolated units fell into one of two classes, with no overlap: a “direct” class consisting of units that followed 5 Hz light stimulation; and a “synaptic” class consisting of units that failed to follow 5 Hz (Figure 5C). This interpretation is further supported by experiments in which we validated the functional test (*i.e.* stimulation at 5 Hz) with pharmacology. All units (N = 5) which continued to respond after NBQX application fell into the direct class. Note that first spike latency did not distinguish direct from synaptic activation (*data not shown*). Since latency would be expected to depend on ChR2 expression levels, the amount of light penetrating, the cell type and other factors that vary from one penetration to the next, this result is not unexpected. In summary, we conclude that the ability to follow reliably during high-frequency stimulus trains can reliably distinguish activation of ChR2-tagged neurons in this system.

Finally, to test whether ChR2-tagged callosally-projecting neurons differed in their auditory responsiveness from nearby untagged neurons selected randomly, we assessed the neural response elicited in each population by white noise bursts. ChR2-tagged neurons were much less responsive to acoustic stimulation than nearby untagged neurons (Figure 5D). Sound-evoked responses in tagged neurons (median 2.4 Hz, N = 21) were lower than in untagged neurons (45.1 Hz, N = 22); spontaneous firing rates were higher in the untagged population (median 4.1 Hz, N = 22) than in the tagged population (median 0.5 Hz, Mann-Whitney U test p = 0.001, N = 21). Thus the population of callosally-projecting neurons appeared to less responsive than nearby untagged neurons.

## Discussion

We have developed PINP, a method for identifying defined neuronal populations *in vivo* using blind extracellular recording techniques. Neurons expressing the ChR2-tag responded with action potentials to brief light flashes, and were identified using conventional extracellular recording. We demonstrate the feasibility and generality of this method by tagging two populations of neurons in the auditory cortex: parvalbumin expressing interneurons, and pyramidal neurons in layers 3 and 5 that project to the contralateral auditory cortex.

ChR2 has emerged as a powerful tool since its recent introduction into neuroscience [17], [29], [30]. Like earlier genetically-encoded neuronal phototriggers [31], [32], [33], ChR2 can be used to assess directly the role of circumscribed neuronal populations in behavior [16], [34], [35], and can be used to map neuronal circuits [30], [36], [37]. ChR2 has also been used to induce plasticity at defined synapses [38]. However, the present use of a phototrigger like ChR2 as a neuronal tag is novel. Previous approaches for tagging neurons *in vivo* have been based mainly on imaging methods—typically two photon microscopy—for recognizing GFP expression or application of calcium dependent indicators, which can be expressed in specific cells types and allow the observation of activity in specified neuronal populations [12], [39]. Though powerful, *in vivo* imaging approaches are technically demanding, particularly in the awake animal, and generally limited to surface brain structures (*but see* [*40*]). By contrast, identification of ChR2-tagged neurons *in vivo* is technically straightforward, requiring little more than an optical fiber, and so can be routinely used in conjunction with conventional extracellular recording.

As with any method, the approach we have described is subject to both false negatives (*e.g.* classifying a parvalbumin-expressing interneuron as PV-negative) and false positives (*e.g.* classifying a PV-negative interneuron as PV-positive). False negatives can arise in several ways: the virus could fail to infect the target neuron; ChR2 expression could be too low, or light too dim, to trigger an action potential. One potential source of false positives is polysynaptic activation. The risk of polysynaptic activation increases with the density of ChR2 expression, and in circuits consisting of excitatory neurons with many recurrent connections. In our cortical preparation, the failure of neurons driven by polysynaptic activation to follow reliably trains of stimuli in excess of 5 Hz allowed us to differentiate tagged and untagged neurons (Figure 5). With the recent introduction of ChiEF [41], a ChR2 mutant with reduced inactivation, this strategy should be even more effective because ChiEF-tagged neurons would be expected to follow flash trains at even higher stimulation rates. Whether this is a general strategy applicable to non-cortical areas remains to be determined. Polysynaptic activation is much less a concern when the tagged neuronal population consists of inhibitory neurons (Fig. 4), since activation of inhibitory neurons suppresses polysynaptic pathways. In the case of cre-mediated excision of the lox-stop-lox cassette, the relatively small number of false positives can be further reduced using newly developed methods that rely on more sophisticated double recombination schemes [35]. The likely prevalence of false negatives over false positives suggests an experimental design in which two different populations of tagged neurons are compared, rather than one in which a tagged population is compared to an un-tagged population.

We used two strategies to tag neuronal populations. One strategy, based on HSV1-mediated retrograde expression of ChR2, is analogous to antidromic electrical stimulation, a classical technique that has revealed functional organization in a number of neural circuits [8], [9]. However, the present variant has some advantages, most notably that the antidromically activated population can be quantified histologically on the basis of YFP labeling. The LSL-AAV strategy, based on the expression of cre-recombinase, is more general, and can be used in any of the growing number of available cre mouse lines.

In the course of these experiments we made two intriguing observations. First, we found that sound-evoked responses in PV-positive neurons were similar to those in nearby untagged neurons. We initially found this surprising. Parvalbumin-expression is found in a class of “fast-spiking interneurons,” which might suggest that this class spikes at high rates. However, the term “fast-spiking” was coined [42] to describe the response of these neurons in acute cortical slices to electrical current injection, and need not imply anything about the responses of these neurons to sensory stimulation. Although under some conditions narrow-spiking presumed PV-positive neurons do respond at higher rates [5], in the auditory cortex the difference between PV-positive and PV-negative responsiveness can be rather subtle [43]. We also found that spontaneous firing rates were higher in untagged neurons, but because the usual sampling bias of extracellular recording toward neurons with high firing rates and large spikes was reduced in searching for flash-evoked responses in ChR2+ neurons, spontaneous firing rates in the two populations may not be directly comparable. Overall, our results indicate that the responses to white noise bursts of the population of so-called fast-spiking interneurons are not qualitatively different from responses in the overall population.

Second, we found that callosally-projecting neurons in auditory cortex were less responsive to simple stimuli than are nearby untagged neurons. This observation is in agreement with previous results indicating that callosally-projecting layer 5 neurons in rat auditory cortex receive more inhibitory input, and have more diffuse receptive fields, than layer 5 neurons that do not project callosally [44], [45]. This observation is also in accord with recent results comparing callosally-projecting (layer 3) neurons with non-projecting neurons in layer 2 (Oviedo and Zador, *unpublished*). Taken together, our results with PV and callosally-projecting neurons suggest that ChR2-tagging can be a powerful approach.

Tagging neurons with ChR2 provides a convenient mean for *in vivo* physiologists to exploit the growing list of methods available for restricting gene expression to defined neuronal populations. This allows us to probe systematically the properties of defined populations of interest, like the parvalbumin expressing interneurons or the callosally projecting neurons in cortical layers 3 and 5. For example, the receptive fields of L5 cells have been investigated and several studies have tried to correlate the recorded activity patterns with the anatomical properties of subgroups of L5 pyramidal neurons [44], [45]. However, these studies were based on slice or anesthetized animal preparations which are not appropriate for investigating neuronal properties in a realistic setting (e.g. in a behaving animal) and provide very low yield. The strategy reported in this study has the advantage of being compatible with other routinely used methods (fiber optics of very small dimensions can be coupled to standard extracellular recording devices, for example [36]) and large portions of brain tissue can be probed in behaving animals with a higher yield, opening a new window to investigate the properties of neuronal circuits.

## Methods

### Viral construction and production

The ChR2-YFP coding region was isolated from pYLECT (kind gift from Karl Deisseroth). To produce AAV, the ChR2-YFP coding region was blunt cloned into the AAV shuttle vector (kind gift of Sandy Kuhlman, [19]) containing a transcriptional insulator flanked by two loxP sites (loxP-STOP-loxP - LSL) downstream of the cytomegalovirus (CMV) promoter. To produce HSV1, the ChR2-YFP coding region was blunt cloned downstream of the elongation factor-1a (EF1a) promoter into the HSV1 shuttle vector obtained from BioVex (London, UK, http://www.biovex.com). Both plasmids were verified by sequencing. High-titer stock of AAV-LSL-ChR2-YFP expressing virus (∼10^12^ pfu/ml in PBS, serotype 2/1) was produced at the Penn Vector Core (University of Pennsylvania) and high-titer stock of the HSV1-ChR2-YFP expressing virus (∼10^10^ pfu/ml in DMEM) was produced by BioVex.

### Viral injection

All procedures were done in accordance to the National Institutes of Health guidelines as approved by the Cold Spring Harbor Animal Care and Use Committee. For AAV injection, male PV-Cre mice (1–2 month old) were anesthetized with Ketamine/Medetomidine ( 120 mg/kg Ketamine,0.5 mg/kg Medetomidine) and positioned in a stereotaxic apparatus. To minimize damage to the injected area of auditory cortex (since the recordings are performed in the injected area), we performed the injections from the top of the brain, opening a craniotomy over the visual cortex (2.3 mm posterior to Bregma, 4.5 mm left from the midline, Mouse Brain Atlas). During the entire procedure animals were kept on a heating pad. The virus (1–2 µl into a single injection site) was delivered with a glass micropipette by pressure injection (20 psi, controlled by a Picospritzer II, General Valve, Fairfield, NJ, USA). The injection was done as follows: the needle was lowered down to 1.5 mm from the pial surface and ∼100 nl were injected, at 40–100 nl/min; the needle was then retracted 50–100 µm and the procedure was repeated. After injection, the craniotomy was covered with silicone sealant, the skin was repositioned with stitches and the animals were returned to their home cages after regaining movement. For the HSV1 injections, male Long-Evans rats (postnatal day 21–30) were anesthetized with Ketamine/Medetomidine (60 mg/kg Ketamine, 0.5 mg/kg Medetomidine). Animals were positioned in a nasorbital stereotaxic apparatus (which allows rotation of the animal's head) and a small craniotomy (1 mm^2^) and durotomy were performed above the primary auditory cortex. The procedure was very similar to the mouse surgery, the animals were kept in a heating pad and the virus was delivered (∼1.5 µl distributed over 6–9 injection sites at 10 nl/pulse, 4 pulses/minute, 150–200 nl per injection site), using the same apparatus. The injections were performed down to 1 mm from the pial surface and hence reached all of the cortical layers, but not the white matter. After injection, the animals were treated as described above.

### Histology

AAV-LSL-ChR2-YFP injected mice and HSV1-ChR2-YFP injected rats were deeply anesthetized and perfused with cold 0.9% solution of NaCl and 4% paraformaldehyde (PFA); the brains were removed and fixated in PFA overnight at 4°C. Coronal sections, 100 µm thick, were prepared with a vibratome (Leica, VT100). Free floating sections from injected mice were permeabilized/blocked in 5% normal goat serum at room temperature for two hours and incubated with anti-parvalbumin antibody (mouse monoclonal, 1∶1000; Sigma, St. Louis, MO) overnight at 4°C. After washing, the sections were incubated for two hours at room temperature with a fluorescent secondary antibody (Alexa594-conjugated goat IgG; 1∶400, Molecular Probes, Eugene, OR), mounted on slides with VectaShield mounting media (Vector Laboratories, Burlingame, CA) and coverslipped. Labeling specificity was assessed by confocal microscopy; independent images for the two channels (YFP and Alexa-594) were acquired and YFP positive cells were then analyzed for co-localization of red fluorescence. Expression levels of the transgene (assessed by YFP visualization) peaked at 2 weeks post-injection and stayed constant for at least 2 months (longer time points were not assessed). Free floating sections from injected rats were permeabilized in 0.1% Triton X-100 and then counterstained with a fluorescent Nissl stain (1∶100, NeuroTrace 530/615, Molecular Probes, Eugene, OR). Sections were washed in phosphate buffered saline, mounted on glass slides and coverslipped with mounting medium. Images were acquired on standard fluorescence microscope. Expression levels of the transgene (assessed by YFP visualization) peaked 10 days post-injection and started dropping after one month. This is probably due to silencing of the viral genome by the host neuron, and not to cell death, since several studies report expression of HSV1 specific latency-associated transcripts well after the expression of reporter genes is lost [46] (Preston 2000). Some tissue damage and cell death were observed exclusively at the site of injection, probably due to the high titer of the virus injected, mechanical damage due to the injection procedure or to an immune response triggered by the presence of antigens in the medium used to re-suspend the viral particles.

### In vivo light stimulation and electrophysiology

Animals were anesthetized with Ketamine/Medetomidine (2 weeks to 2 months post-injection for mice, and 10 to 20 days for rats) and positioned in a custom naso-orbital restraint. A cisternal drain was performed, and a craniotomy/durotomy was performed above the primary auditory cortex, on the same side of the injection site for mice and on the contralateral site for rats. An LED coupled to a fiber optic (LED Pigtail Luxeon III Star-blue, 473 nm, 1 mm diameter, 17 mW absolute power, Doric Lenses, Quebec, Canada) was positioned above the exposed surface of the brain with the aid of a micromanipulator. The LED was controlled and all data were obtained using a custom data acquisition system written in Matlab (Mathworks, Natick, MA).

All experiments were conducted in a double-walled sound booth (Industrial Acoustics Company, Bronx, NY). Free-field acoustic stimuli were presented at a 200 kHz sampling rate using a custom real-time Linux system driving a high-end Lynx L22 audio card (Lynx Studio Technology Inc., Newport Beach, CA) connected to an amplifier (Stax SRM 313, STAX Ltd, Japan), which drove a calibrated electrostatic speaker (taken from the left side of a pair of Stax SR303 headphones) located 8 cm lateral to, and facing, the contralateral (right) ear. For stimulation of the mouse PV-positive neurons we used LED pulses (10 ms) and white noise bursts (35 ms) delivered at 0.67 Hz. For stimulation of the rat pyramidal neurons we used LED pulses (3–10 ms) and clicks (5 ms) with inter-stimulus intervals of 500, 200, 100, 50, 30, and 20 ms. Spike responses were recorded extracellularly using tungsten electrodes (mouse: 1–3 MΩ World Precision Instruments, Sarasota, FL) or quartz-coated platinum/tungsten tetrodes (rat: 1–2 MΩ Thomas Recording, Germany), with a sampling rate of 32 kHz. Recorded signals were passed through AI-401 headstage and then further amplified and band-pass filtered at 0.3―6 kHz high using a CyberAmp 380 amplifier (Axon Instruments, Union City, CA).

### Pharmacology

For blockage of synaptic transmission in rat auditory cortex, a 1.0 mM NBQX solution in a physiological buffer was applied to the surface of the cortex while playing 5 ms, 70 dB, white noise stimuli and monitoring the local field potential (LFP) recorded with the quartz-coated platinum/tungsten tetrode, 500 µm below the cortical surface. Extracellular recordings and LED stimulation were only attempted after complete abolition of all evoked and spontaneous LFP responses.

### Data analysis

Recorded spikes were extracted from raw voltage traces by applying a high-pass filter and thresholding. Spike times were then assigned to the peaks of suprathreshold segments. For tetrode recordings, 1 ms spike waveforms were extracted from each recorded (high-passed) channel, and single units were identified after clustering using MClust and KlustaKwik (Kenneth Harris, klustakwik.sourceforge.net). We used spike peak, spike valley and spike energy as the main parameters for clustering. Stimulus evoked responses were computed as spike counts in 40 ms windows after stimulus onset. Response reliability was computed as fraction of trials in which the firing rate in response window was greater than firing rate in a corresponding time window immediately preceding stimulus onset. For analysis of multiunit recordings in Figure 5, only unclustered spikes were considered.
